# A closer look at the role of urotensin II in the metabolic syndrome

**DOI:** 10.3389/fendo.2012.00165

**Published:** 2012-12-28

**Authors:** Pierre-Olivier Barrette, Adel Giaid Schwertani

**Affiliations:** Division of Cardiology, Department of Medicine, McGill University Health CenterMontreal, QC, Canada

**Keywords:** metabolic syndrome, insulin resistance, inflammation, obesity, dyslipidemia, hypertension, diabetes

## Abstract

Urotensin II (UII) is a vasoactive peptide that was first discovered in the teleost fish, and later in mammals and humans. UII binds to the G protein coupled receptor GPR14 (now known as UT). UII mediates important physiological and pathological actions by interacting with its receptor. The metabolic syndrome (MetS) is described as cluster of factors such as obesity, dyslipidemia, hypertension, and insulin resistance (IR), further leading to development of type 2 diabetes mellitus and cardiovascular diseases. UII levels are upregulated in patients with the MetS. Evidence directly implicating UII in every risk factor of the MetS has been accumulated. The mechanism that links the different aspects of the MetS relies primarily on IR and inflammation. By directly modulating both of these factors, UII is thought to play a central role in the pathogenesis of the MetS. Moreover, UII also plays an important role in hypertension and hyperlipidemia thereby contributing to cardiovascular complications associated with the MetS.

## UROTENSIN II

The peptide Urotensin II (UII) was first identified in the teleost fish *Gillichthys mirabilis* (Pearson et al., 1980). Homologs of this peptide were subsequently found in mammals and humans (Pearson et al., 1980; Conlon et al., 1996; Ames et al., 1999; Coulouarn et al., 1999). Although the amino acid sequence of these homologs changes depending on the species, a cyclic sequence of six amino acids is conserved (Cys-Phe-Trp-Lys-Tyr-Cys). It is believed that this sequence is responsible for the biological activity of the peptide (Conlon et al., 1990). UII acts as a vasoactive peptide by binding to the G protein coupled receptor GPR14, better known as UT (Ames et al., 1999; Liu et al., 1999). A recent study has shown that UII vasoconstrictive effects are mediated by calcium influx via STIM1 and Orai-1 (Domínguez-Rodríguez et al., 2012). Human UII (hUII) and its receptor have been found in cardiac and vascular tissues, spinal cord, central nervous system, kidney, liver, and pancreas (Maguire et al., 2000; Matsushita et al., 2001). UII is also present in the blood plasma in picomolar concentrations (Matsushita et al., 2001). However, it tends to act mainly in an autocrine and paracrine fashion rather than as a hormone (Yoshimoto et al., 2004). Even though the relation between UII and its receptor was made in 1999, the physiological and pathological roles of UII are only beginning to be unraveled. In a healthy state, the binding of UII with UT is known to play an important role in the control of vascular tone, blood pressure, and insulin release (Douglas and Ohlstein, 2000; Douglas et al., 2000; Loirand et al., 2008). UII has been identified as the most potent vasoactive peptide known to date, being more potent vasoconstrictor than endothelin-1 and angiotensin-II (Ames et al., 1999; Douglas and Ohlstein, 2000; Maguire et al., 2000). UII also controls the function of vascular smooth-muscle cells (VSMCs) through the release of endothelial-cell-derived vasodilators such as nitric oxide (Gibson, 1987; Gardiner et al., 2001; Stirrat et al., 2001). The role of UII in pathological states is still debated. Many studies have shown increased levels of UII and its receptor in diverse cardiovascular and metabolic diseases such as type 2 diabetes mellitus, renal dysfunction, atherosclerosis, systematic and essential hypertension, obesity, congestive heart failure, myocardial infarction, cardiac fibrosis, hypertrophy, and remodeling (Reviewed in Hassan et al., 2003; Ross et al., 2010; Gruson et al., 2012). The elevated levels of UII in disease states suggest that UII is expressed either as a protective response to pathologies or as a pathological agent. Some studies claim that temporary elevation of UII levels may lower cardiovascular disease risk factors in end-stage renal disease (Mallamaci et al., 2005; Zoccali et al., 2006), myocardial infarction (Babińska et al., 2012) and restore endothelial function (Zoccali and Mallamaci, 2008). On the other hand, other studies have demonstrated that UII acts as a pathological agent inducing cardiac hypertrophy in synergy with angiotensin II by phosphorylation of the Akt kinase (Chanalaris et al., 2005; Gruson et al., 2010b, 2012).

## METABOLIC SYNDROME

As mentioned above, the metabolic syndrome (MetS) consists of a set of risk factors, such as obesity, hyperlipidemia, hypertension, hyperglycemia, and insulin resistance (IR), leading to the development of type 2 diabetes, cardiovascular diseases, non-alcoholic fatty liver disease, and renal impairment (Grundy, 1999; Cho, 2011). The relation between these risk factors and complications seems to be mostly attributed to IR (Eckel et al., 2010), which is why the syndrome was first defined as an IR syndrome (Reaven, 1988). Since then, the National Cholesterol Education Program (NCEP)-Adult Treatment Panel III, the World Health Organization (WHO) and the International Diabetes Federation (IDF) set up some criteria for the clinical definition of MetS.

A recent study using data from the 2003–2006 National Health and Nutrition Examination Survey and the NCEP/IDF MetS criteria, revealed that the prevalence of MetS was 34.3% among all adults in the US (Ford et al., 2010). However, high prevalence of MetS is not only restricted to the Western world. MetS seems to be highly prevalent in sub-Saharan Africa, Middle-East, South America, and South Asian Countries (Misra and Khurana, 2008). Central obesity is considered the main precursor to MetS (Cameron et al., 2008). According to the WHO, the global obese population increased from 200 million in 1995 to 300 million in 2000. The WHO has also determined that the obesity epidemic is also spreading in developing countries with over 115 million people suffering from obesity-related illnesses (Misra and Khurana, 2008; WHO, 2008). Popkin and Doak (1998) have also reported increasing obesity prevalence in the United States, Latin America, Europe, and Asia, which confirms the worldwide epidemic proportions of obesity and overweight. In addition, the Aerobic Center Longitudinal Study, which included 33,230 cancer-free men followed for 14 years, showed 56% enhanced risk of cancer mortality for men affected by MetS (Jaggers et al., 2009). The direct implication of the syndrome in CVD, type 2 diabetes, non-alcoholic fatty liver, and cancer, as well as an increasing prevalence of obesity and decreased physical inactivity in Western societies makes the MetS a major health preoccupation (NCEP, 2002)

Recent researches have shown that UII has an impact on the risk factors as well as the overall pathogenesis of the MetS (Ong et al., 2008). A study using the American Heart Association/National Heart, Lung, and Blood Institute criteria for MetS revealed that MetS patients show higher plasma levels of UII (Gruson et al., 2010a). Our group also demonstrated an increase in UII plasma levels in mice when all risk factors of MetS are present (You et al., 2012). Moreover, we recently found that UII gene deletion in mice (UIIKO) significantly decreased body mass, visceral fat, blood pressure, and increased insulin and glucose tolerance when compared to wild-type mice (You et al., 2012). Even though there is a clear association between MetS and UII, it is still unclear whether the peptide plays a role in the initiation of the disease, or if the elevated plasma levels of UII are a result of the syndrome. This review aims to give a closer look at the implication of UII in the risk factors and pathways involved in the development of the MetS.

## INSULIN RESISTANCE (IR)

Insulin plays a major role in glucose and lipid metabolism such as adipose tissue triglyceride lipolysis, lipoprotein lipase activity, muscle and adipose tissue glucose absorption, muscle and liver glycogen synthesis, and endogenous glucose production (Cornier et al., 2008). When cells react poorly to an insulin stimulus, they are defined as IR. The latter is known to be the link between the individual components of MetS (Eckel et al., 2010). For instance, it seems that IR correlates positively with atherosclerosis (Fernández-Real and Ricart, 2003) and coronary artery disease (CAD; Juhan-Vague et al., 1991), and also an indirect cause of hyperinsulinemia (Reaven, 2003), dyslipidemia, hyperglycemia, and hypertension (Sowers, 2004) through an increase in free fatty acid (FFA) synthesis in adipose tissue (Boden and Shulman, 2002) and a chronic inflammatory response (Fernández-Real and Ricart, 2003).

The IR Atherosclerosis Study (IRAS) and the Atherosclerosis Risk in Communities Study showed that insulin sensitivity (measured with an intravenous glucose tolerance test) correlates negatively with intimal-medial thickness of the carotid artery (Howard et al., 1996). Subsequent studies also found a significant correlation between carotid intimal-media thickening and IR and hyperinsulinemia (Golden et al., 2002; Zavaroni et al., 2006). Intimal-media thickness has been shown to be a significant predictor of myocardial infarction and coronary death (Hodis et al., 1998). As a result, IR is a major risk factor in developing cardiovascular complications. Indeed, IR has been associated with increased plasma levels of plasminogen activator inhibitor 1 (PAI-1; Juhan-Vague et al., 1991). Increased levels of PAI-1 are known to impair fibrinolysis, which leads to progression of coronary atherosclerosis in glucose intolerant patients (Bavenholm et al., 1998) and increased risk of myocardial reinfarction within 3 years in less than 45-year-old men (Hamsten et al., 1985, 1987; Gram et al., 1987; Juhan-Vague et al., 1991) as well as increased risk of CAD (Juhan-Vague et al., 1991). UII is known to increase expression of PAI-1 in vascular SMC (Djordjevic et al., 2005), hence UII may contribute to atherosclerosis through PAI-1 inhibition of fibrinolysis.

Free fatty acids play an important role in MetS by having direct effects on dyslipidemia, hypertension (Fagot-Campagna et al., 1998), IR (Boden, 1997), and pancreatic β-cell dysfunction (Sako and Grill, 1990; Unger, 1995; McGarry and Dobbins, 1999; Boden and Shulman, 2002). Impaired insulin action in adipose tissue tends to increase FFAs concentrations in the plasma by reducing the insulin inhibition of adipose tissue triglyceride lipolysis (Randle et al., 1963; Randle, 1998; Kahn and Flier, 2000; Cornier et al., 2008). On the other hand, FFAs induce IR in skeletal muscles (DeFronzo et al., 1985) and liver by inhibiting insulin suppression of glycogenolysis (Boden et al., 2002), which results in a vicious cycle increasing IR, FFA levels, and promotion of hyperglycemia. Furthermore, higher plasma glucose and FFA levels tend to increase insulin secretion in the pancreas (Boden and Shulman, 2002), thus leading to hyperinsulinemia. Such high plasma insulin levels promote hypertension (Welborn et al., 1966; Reaven, 2003), which is another risk factor of MetS. An increase in FFA levels also predicts development of type 2 diabetes (Paolisso et al., 1995; Charles et al., 1997). In the liver, higher FFA levels increase low density lipoprotein (LDL) and triglyceride production, while lowering high density lipoprotein (HDL) production (Brinton et al., 1991; Muramaki et al., 1995).

The direct effects of UII on lipid mobilization and FFA release are somewhat contradictory. In the coho salmon, administration of UII stimulated the activity of triacylglycerol lipase and the release of FFAs in the liver (Sheridan and Bern, 1986; Sheridan et al., 1987). On the contrary, UII injection in dogfish showed no significant increase in FFA release or plasma triglyceride concentrations (Conlon et al., 1994). We recently found that UII gene deletion in mice fed high fat diet significantly reduced serum levels of FFAs in comparison with wild-type (You et al., 2012). In light of our own findings and those of others linking UII to IR and glucose metabolism (Silvestre et al., 2001, 2004; Ong et al., 2006; You et al., 2012), we suggest that UII may mediate lipid mobilization and FFA indirectly through IR. However, the direct influence of UII in lipid metabolism still requires further research.

**FIGURE 1:**
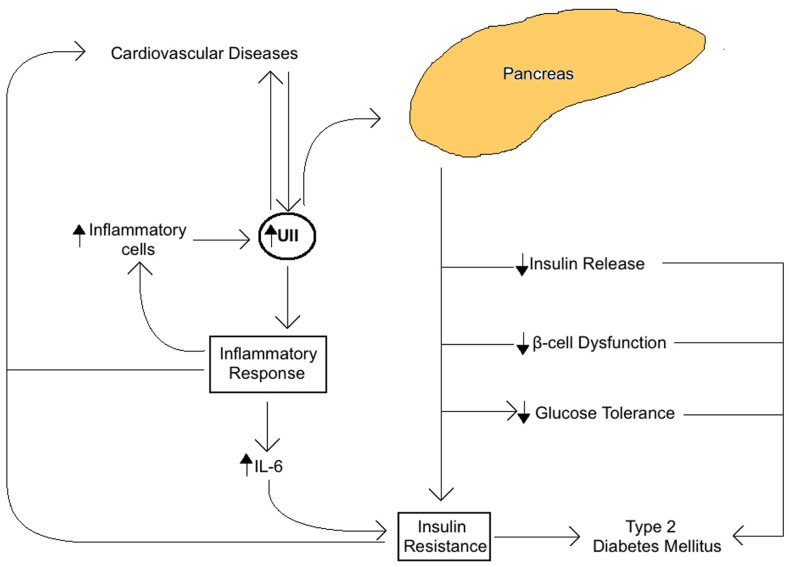
**Summary of the role of Urotensin II (UII) on insulin activity and glucose metabolism associated with the metabolic syndrome**. UII expressed in the pancreas acts directly to impair glucose tolerance, reduce glucose-mediated insulin release, impair pancreatic β-cell function, and block insulin response resulting in IR and increased risk of type 2 diabetes and cardiovascular diseases.

The role of UII in the MetS seems to be closely related to insulin activity and the overall glucose metabolism in the pancreas (**Figure 1**). In fact, UII and UT are both expressed in rat pancreatic islets, where their interaction seems to inhibit glucose-mediated insulin release (Silvestre et al., 2001, 2004). Also, when high concentrations of UII are administrated to the rat pancreas, glucose and arginine induced insulin response are blocked (Silvestre et al., 2001, 2004). Some UII and UT gene haplotypes are known to be associated with IR (Ong et al., 2006), impaired glucose tolerance (Ong et al., 2006), β-cell pancreatic function, and type 2 diabetes (Sun et al., 2002; Zhu et al., 2002; Wenyi et al., 2003; Suzuki et al., 2004; Tan et al., 2006; Sáez et al., 2011). UII upregulation in plasma and skeletal muscle in type 2 diabetes mellitus is coherent with this association (Totsune et al., 2003; Wang et al., 2009). In addition, UII increases glucose-6-phosphatase activity in salmon liver, which tends to reduce glycogen content and increase glucose levels, leading to hyperglycemia. Our recent study has demonstrated that UII gene knockout in mice reduced serum glucose and insulin, and increased glucose and insulin tolerance in comparison with wild-type mice (You et al., 2012).

## INFLAMMATORY RESPONSE IN THE MetS

It is well known that IR and MetS are both associated with an inflammatory response (Sutherland et al., 2004). The relationship between IR and inflammation seems to be bidirectional; IR promotes an inflammatory response and vice versa (Fernández-Real and Ricart, 2003), resulting in a vicious circle increasing risk of MetS incidence. An acute-phase inflammatory response is not only linked with IR (Fernández-Real et al., 2001; Festa et al., 2002; Grimble, 2002; Leinonen et al., 2003), but also with type 2 diabetes (McMillan, 1989; Arnalich et al., 2000; Festa et al., 2002; Leinonen et al., 2003), obesity (Laimer et al., 2002; Leinonen et al., 2003), and the overall MetS (Chan et al., 2002; Das, 2002). The activity of inflammatory markers such as C-reactive protein, tumor necrosis factor-alpha (TNF-α), Interleukin-18 (IL-18) and PAI-1 increases as the presence of MetS risk factors increase (Cornier et al., 2008).

Tumor necrosis factor-alpha prevents the action of insulin in cultured cells and in animal models (Hotamisligil et al., 1993, 1994, 1996) by inducing serine phosphorylation of the insulin receptor substrate-1, which reduces the tyrosine kinase activity of the insulin receptor (Hotamisligil et al., 1996), resulting in IR. Furthermore, TNF-α stimulates the production of endothelin-1 (Kahaleh and Fan, 1997) and angiotensin (Brasier et al., 1996) *in vitro*, which are directly involved in vasoconstriction and thus may lead to increased hypertension. Indeed, there is a positive correlation between TNF-α, systolic blood pressure, and IR (Dörffel et al., 1999). TNF-α also stimulates VLDL production (Grunfeld and Feingold, 1992) and decreases HDL cholesterol (Jovinge et al., 1998). Dyslipidemia in mice is significantly related to increased TNF-α levels (Fleet et al., 1992).

Elevated levels of the inflammatory cytokine IL-6 are associated with obesity, IR (Fried et al., 1998; Bastard et al., 2000; Fernández-Real et al., 2001; Kern et al., 2001; Bastard et al., 2002), and type 2 diabetes (Pradhan et al., 2001; Vozarova et al., 2003). IL-6 also plays a role in the pathology of dyslipidemia by decreasing LPL activity in adipocytes (Greenberg et al., 1992) which increases hepatic triglyceride levels (Nonogaki et al., 1995). In man, IL-6 correlates with increased FFA (Stouthard et al., 1995), fasting triglycerides, and VLDL (Fernández-Real et al., 2000), and decreased HDL cholesterol (Zuliani et al., 2007). This cytokine may also have an impact on hypertension by stimulating the sympathetic and central nervous system (Besedovsky and Del Rey, 1996; Papanicolaou et al., 1996), and angiotensin expression (Takano et al., 2000).

**FIGURE 2:**
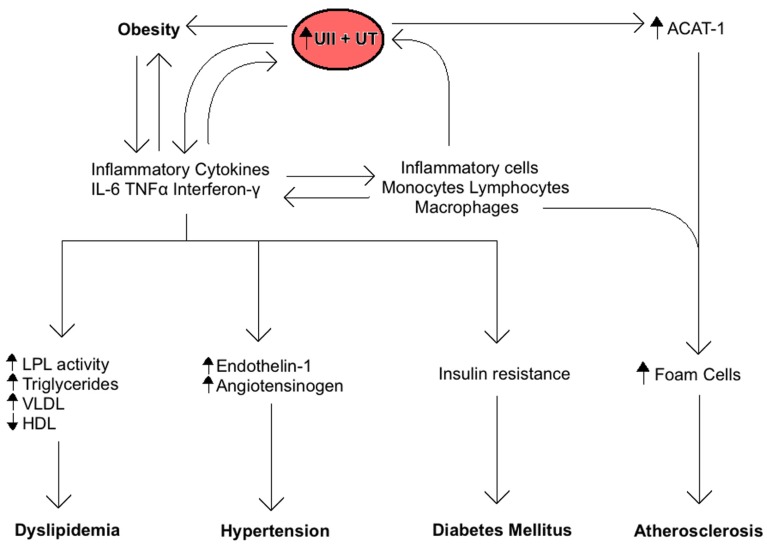
**Summary of the role of Urotensin II (UII) on the inflammatory response associated with the metabolic syndrome**. Inflammatory cytokines increase UII and UT expression and vice-versa, resulting in a positive feedback. The ensuing chronic inflammation is directly involved in the development of obesity, insulin resistance, dyslipidemia, hypertension, and atherosclerosis.

The central role of the inflammatory response in MetS is also related to UII and UT expression (**Figure 2**). Inflammatory cells including lymphocytes, macrophages, monocytes, and foam cells express UII and UT mRNA (Bousette et al., 2004). Lymphocytes are by far the largest producers of UII, whereas monocytes and macrophages produce the highest levels of UT. Moreover, inflammatory markers such as TNF-α, lipopolysaccharide, and interferon-γ are all known to induce UT expression *in vitro* (Segain et al., 2007). These findings suggest that an inflammatory response to MetS and its components may increase UII levels by increasing inflammatory cells production of the peptide. On the other hand, UII is known to contribute to the inflammatory response by inducing the release of inflammatory cytokines like IL-6 in cardiomyocytes (Sano et al., 2000; Tzanidis et al., 2003; Johns et al., 2004; Russell and Molenaar, 2004). UII mediates aortic inflammation by stimulating the release of leukotriene C (LTC_4_), a lipid inflammatory mediator, from aortic adventitial fibroblasts through 5-lipoxygenase pathway (Dong et al., 2012). LTC_4_ plays a key role in the pathogenesis of atherosclerosis as a chemoattractant and activator for monocytes in the vascular endothelium. It is also known to initiate contraction of smooth muscle cells in the adjacent medial tissues of the vasculature (Dong et al., 2012). In addition, we have recently shown that UII gene deletion in mice fed a high fat diet reduces the serum levels of inflammatory cytokines including monocyte chemoattractant protein-1, monokine induced by γ-interferon, and keratinocyte chemoattractant when compared to wild-type mice (You et al., 2012).

## UII, HYPERTENSION, AND CARDIOVASCULAR DISEASES

The MetS is identified as a clustering of risk factors that perpetuate cardiovascular diseases. Since UII has been shown to play a role in the pathogenesis of atherosclerosis (Maguire et al., 2004; Hassan et al., 2005; Papadopoulos et al., 2009), congestive heart failure (Hassan et al., 2003), myocardial ischemia (Zhang et al., 2002; Zhou et al., 2003), ventricular hypertrophy, and fibrosis (Sano et al., 2000; Tzanidis et al., 2003; Johns et al., 2004; Russell and Molenaar, 2004), it is therefore reasonable to suggest that induction of UII in the MetS may contribute to the cardiovascular abnormalities associated with this syndrome.

Atherosclerosis is the main cause of morbidity and mortality in cardiovascular diseases and cause of death in the Western world (Libby et al., 2009). Atherosclerosis is known to induce UT and UII overexpression in mice aortic tissues (Douglas et al., 2002; Bousette et al., 2004; Wang et al., 2006; Libby et al., 2009). In mice overexpressing UT receptor (UT^+^), aortic atherosclerosis lesion formation is significantly increased (Papadopoulos et al., 2009), whereas lesion formation is decreased in UIIKO mice with an atherosclerotic background (APOE; You et al., 2012). UII is known to promote vascular remodeling by mediating VSMC proliferation and migration inside the intima, a process directly involved in atherogenesis (Sauzeau et al., 2001; Watanabe et al., 2001a,b; Tamura et al., 2003; Albertin et al., 2009; Iglewski and Grant, 2010). It has been suggested that vascular endothelial growth factor (VEGF), a major angiogenic protein, could be responsible for UII-mediated vascular remodeling. Even though UII does not affect VEGF expression in human endothelium (Albertin et al., 2009), recent evidence has shown that UII induces secretion of VEGF in the adventitia in synergy with angiotensin II (Song et al., 2012). VEGF stimulates proliferation of endothelial and smooth muscles cells while also stimulating proliferation and migration of adventitial fibroblasts in the intima (Sartore et al., 2001; Stenmark et al., 2006; Zhang et al., 2008). In addition, UII contributes to the inflammatory response in atherosclerosis by acting as a chemoattractant for monocytes (Segain et al., 2007) and increasing IL-6 expression in cardiomyocytes (Sano et al., 2000; Tzanidis et al., 2003; Johns et al., 2004; Russell and Molenaar, 2004). UII interacts with UT receptors on the surface of macrophages to promote expression of ACAT-1 (Watanabe et al., 2005). ACAT-1 is known to accelerate foam cells formation, which has a major impact on atherosclerotic lesion development (Watanabe et al., 2005). Another main effect of UII on atherosclerosis is the increased expression of NADPH oxidase, which is a main source of reactive oxygen species (ROS; Djordjevic et al., 2005; Tsoukas et al., 2011). ROS are central in the early initiation of atherosclerosis by converting LDL into oxidized-LDL (Djordjevic et al., 2005). NADPH oxidase-derived superoxide inactivates nitric oxide, resulting in impaired endothelial dependant vasodilatation (Zou et al., 2004) and hypertension (Lassègue and Clempus, 2003).

Urotensin II also contributes to essential (Matsushita et al., 2001) and secondary (Heller et al., 2002) hypertension by inducing vascular remodeling. Increased blood pressure in cats (Behm et al., 2004), rats (Lin et al., 2003), and sheep (Watson et al., 2003) with UII administration is coherent with these findings. In humans, plasma UII correlates positively with systolic blood pressure, independently of an atherosclerotic background (Cheung et al., 2004). Indeed, the UII gene (UTS2) is associated with essential hypertension (Yi et al., 2006) and myocardial infarction (Nishihama et al., 2007; Oguri et al., 2009). In a chronic heart failure or essential hypertension state, UII loses its dilatory function (Lim et al., 2004; Sondermeijer et al., 2005).

## UII, OBESITY, AND HYPERLIPIDEMIA

Obesity seems to be a main cause of IR in both men and women (Carey et al., 1996; Cornier et al., 2008; Stefan et al., 2008). Moreover, it seems that physical activity and a diet rich in monounsaturated or hydrogenated fat leads to an impairment of insulin sensitivity (Mayer-Davis et al., 1998; Vessby et al., 2001; Han et al., 2002). Obesity is also known as a chronic inflammatory state; it induces release of proinflammatory cytokines and adipokines (Yudkin et al., 1999; Festa et al., 2001; Engström et al., 2003; Trayhurn and Wood, 2004). As a result, the pathologic role of inflammation in IR and MetS may be enhanced by obesity. However, some suggest that obesity may be, on the opposite, an outcome of inflammation (Das, 2001). Obesity may contribute to increase local IR in the adipocytes as well as in other tissue such as the liver and skeletal muscles (Cornier et al., 2008; Gregor and Hotamisligil, 2011).

**FIGURE 3:**
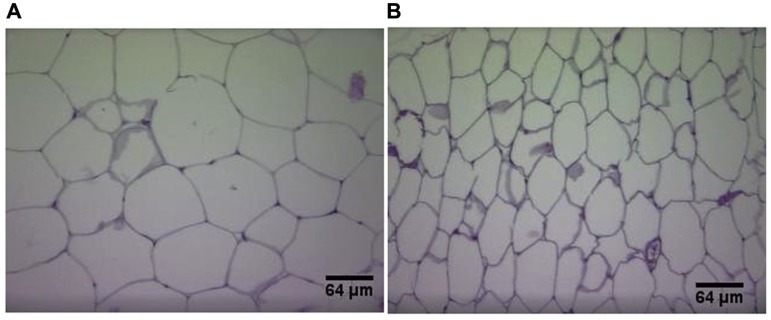
**Hematoxylin and eosin staining of visceral adipose tissue sections in wild-type mice (A) and UIIKO mice (B)**. Adipocytes of UIIKO mice appear smaller than wild-type mice.

Plasma UII correlates positively with body weight in humans independently of atherosclerotic background (Cheung et al., 2004). Adipokines (dipeptidyl peptidase-4, endocan, insulin-like growth factor-binding proteins), known to play a role in diabetes mellitus and obesity (Janke et al., 2006; Neumiller et al., 2010; Ruan and Lai, 2010) were reduced in UII/ApoE double knockout mice in comparison with ApoEKO (You et al., 2012). Reduction of body mass, visceral fat, visceral adipocytes diameter, serum LDL, triglycerides, as well as increase in HDL is also observed with UII gene deletion in mice (You et al., 2012; **Figure 3**). A reduction in hepatic cholesterol esterification is also observed in UIIKO mice (Kiss et al., 2011). These results are attributed to a reduction in cholesterol and apolipoprotein B production by hepatocytes (Kiss et al., 2011). Furthermore, Jiang et al. (2008) found that the UII gene (UTS2) regulates fat accumulation in humans.

## CONCLUSION

The mechanism that links the various components of MetS relies primarily on IR and inflammation (Sutherland et al., 2004; Eckel et al., 2010). The ability of UII to modulate both of these factors suggests an important role for the peptide in the pathogenesis of the MetS. Other risk factors mediated by UII, such as obesity, may tend to initiate the positive feedback cycle, which contributes to the development of MetS. In fact, genetic manipulation or the use of UT receptor antagonists seems to have a positive effect on every risk factor of the MetS (You et al., 2012). However, it is still unclear whether UII is important in the initiation or only in the progression and further complications of the disease. The biological activities of UII, and the recent reports of increased serum levels of UII in patients with the MetS suggest that pharmacological manipulation of the UII pathway as a possible future treatment of the syndrome.

## Conflict of Interest Statement

The authors declare that the research was conducted in the absence of any commercial or financial relationships that could be construed as a potential conflict of interes
